# The Impact of Pressure-Dependent Viscosity Data on Injection Molding Simulations of Highly Filled Thermoplastics

**DOI:** 10.3390/polym17243322

**Published:** 2025-12-16

**Authors:** Felix Kerling, Samuel Schlicht, Benedikt Roth, Tobias Kleffel, Uta Rösel, Dietmar Drummer

**Affiliations:** Institute of Polymer Technology, Friedrich-Alexander-Universität Erlangen-Nürnberg (FAU), Am Weichselgarten 10, 91058 Erlangen, Germany; samuel.schlicht@fau.de (S.S.); benedikt.roth@gmx.de (B.R.); tobias.kleffel@gmail.com (T.K.); uta.ur.roesel@fau.de (U.R.); dietmar.drummer@fau.de (D.D.)

**Keywords:** injection molding, injection compression molding, bipolar plate, fuel cell, thin-wall injection molding, dynamic mold temperature control, pressure dependent viscosity

## Abstract

The injection compression molding using dynamic mold control (ICM-DT) represents a promising technological approach to the manufacturing of highly filled, modified thermoplastic components with tight geometric tolerances. While the numerical prediction of flow states is, to date, predominantly based on the Cross–WLF modeling of viscoelastic characteristics of the melt, new material-related developments necessitate the assessment of process- and material-related boundaries. The present paper employs a highly filled graphite–polypropylene system, exhibiting a graphite mass fraction of 80%, for the quantitative comparison of Cross–WLF predictions and experimentally derived flow states. Based on coupled counter pressure-chamber high-pressure capillary rheometry (CPC-HCR) and counterpressurized viscometry (CPV) alongside the ICM-DT of thin-walled specimens, pressure-induced crystallization was identified to induce significant deviations from Cross–WLF predictions. Cross–WLF modeling strongly overestimates the processability of the applied graphite–polypropylene system under both injection molding (IM) and ICM regimes. We therefore observe a predominant influence of pressure-induced crystallization mechanisms in dynamic mold temperature process domains, in which the pressure-induced, crystallization-related exponential viscosity increase cannot be adequately modeled through both pressure-dependent and pressure-agnostic Cross–WLF models. The numerical approximation of flow states under dynamic mold temperature regimes hence necessitates the consideration of solidification-induced, self-intensifying pressure excursions.

## 1. Introduction

As the global energy transition progresses and the electrification of the transportation sector expands, emission-free propulsion and energy storage solutions are gaining increasing relevance. Polymer electrolyte membrane fuel cell (PEMFC) technology is pivotal in facilitating the commercial use of hydrogen as a potential energy storage medium for medium- and long-distance transportation, including trucks, marine vessels, and aviation, as well as for stationary and decentralized energy supply systems within the broader energy sector [1]. However, for widespread adoption, significant improvements in both the cost-effectiveness and lifespan of PEMFC systems are essential to make them competitive with conventional fossil-fuel-based propulsion and storage technologies [2]. The optimization of stack design and the construction of individual fuel cell components should ideally focus on enhancing both performance and longevity [3], while minimizing the thickness and volume of the components to reduce costs and save space [4]. In addition to these design challenges, the materials used must meet stringent demands. Among the components, the bipolar plate stands out as having the most critical requirements and potential for optimization, as it accounts for 30–40% of the overall cost [5] and up to 80% of the total weight of a fuel cell [6]. Therefore, the bipolar plate must exhibit high electrical conductivity, mechanical stability, and corrosion resistance, while also being lightweight, compact, affordable, and efficiently replicated channel structures for gas distribution [2]. Current research focuses on both metallic and polymer composite materials to meet these demands. Metallic materials offer high electrical conductivity and mechanical strength, flexibility [7], and low component thickness [8]. However, they are prone to corrosion and oxidation due to the reaction products formed during electrochemical processes [9]. This leads to increased contact resistance, reduced power output, and shorter cell lifespans [10], often necessitating costly and complex corrosion-resistant coatings [4]. Current developments include the improvement of corrosion resistance through the use of advanced coatings [11], the improvement of current coating processes [12], the usage of new materials such as titanium [13], and the improvement of current production processes like forming and stamping [14]. Among the coatings, pure carbon coatings [15], Cr-doped a-C coatings [16], conductive polymer coatings [11], transition metal carbides (TMCs) [17], metal nitride and carbide films [18], and precious metal coatings [12] are the most important representatives that are currently the subject of particular attention in research [14]. Moreover, the production of metallic bipolar plates is constrained by either inefficiency and high processing costs [19] or limitations in channel structure complexity, with high aspect ratios frequently causing dimensional errors, cracks, and wrinkles due to substrate thinning [20]. On the other hand, polymer composite-based bipolar plates are emerging as a technically and economically viable alternative. They offer distinct advantages, including reduced specific weight, enhanced corrosion resistance [21], durability [10], high functional integration within complex 3D geometries, and lower production costs [22].

The electrical insulating properties of polymers, which are disadvantageous for this application, can be significantly modified through the incorporation of various fillers and additives into the polymer matrix. The introduction of conductive elements such as fibers, flakes, or spherical particles made from metals, conductive carbon black, graphite, or nanofillers like carbon nanotubes (CNTs) can reduce the resistivity of polymers by several orders of magnitude [23]. Several studies have attempted to improve the electrical conductivity of polymer-based bipolar plates by modifying the filler, using materials such as graphite powder [24] or conductive carbon black in combination with carbon microspheres [25]. Roth et al. showed that with a total filler content of 80 wt.% of graphite flakes [26], improved bipolar plates with excellent electrical conductivity properties can be achieved.

To use the cost and durability advantages of polymers in the production of bipolar plates, it is essential to achieve high filler content to ensure adequate electrical conductivity. However, such modifications to thermoplastic melts lead to significantly increased viscosity and negatively impact melt thermal conductivity, thereby impeding mold filling and replication quality. This, in turn, restricts the achievable thickness and dimensional stability of bipolar plates produced via injection molding [26]. In contrast, metallic bipolar plates are already being produced with thicknesses as low as 100 μm [8], highlighting that the weight benefits of polymer-based plates have yet to be fully utilized.

Given that the output voltage of a single cell is capped at a maximum of 1.23 V, multiple cells must be connected in series to achieve higher voltages. Consequently, thicker polymer-based plates contribute to increased space requirements and higher costs. Reduced plate thicknesses result in a diminished flow cross-section. Higher injection speeds and elevated mold temperatures therefore seem necessary but have proven to be insufficient, as these approaches lead to excessive pressure demands during filling [27] and generate non-uniform pressure and temperature distributions along the flow path [28]. At the component level, these challenges manifest as flow-path-dependent variations in thickness accuracy [29] and reduced precision in molding the intricate channel structures [6].

Rzeczkowski et al. [27] showed with an isothermal mold temperature that the injection molding process is limited to an aspect ratio of 40 with a plate dimension of 80 × 80 × 2 mm^3^. The material that was used was PP with 80 wt.% of graphite. Brokamp et al. [30] achieved an aspect ratio of ~48 with plate dimensions of 142.5 × 80 × 3 mm^3^ and a highly filled PPS graphite compound with a filler content of 75 wt.%. Roth et al. [26] showed that with an injection compression molding (ICM) process with a dynamic mold control (DT), aspect ratios of up to 90 can be achieved. Due to the limitations of the maximum compression stroke of the mold used by Roth et al., it was not possible to further investigate which maximum aspect ratios can be achieved in the ICM-DT process. The literature search did not reveal any other peer-reviewed research activities other than the work of Roth et al. [26] in the field of bipolar plate production by injection compression molding with dynamic mold control.

Numerical investigations represent a frequently applied method for analyzing the maximum achievable ratio of flow path length to wall thickness in more detail. By using simulations, costly and complex iterative processes with physical molds can be avoided. The maximum ratio of flow path length to wall thickness can be determined before the final mold is built. The basis for determining the maximum flow paths and the aspect ratio is the accuracy of the simulation and the material models. Therefore, the usage of pressure-dependent viscosity models is recommended by Wang et al. for simulating parts with high surface-to-volume ratio (S/V) [31].

During processing, small components face different time–temperature–pressure conditions compared to larger parts [32]. The higher surface-to-volume (S/V) ratio of micro- and thin-walled components causes a faster heat transfer between the melt and the mold [33]. As a result, the melt cools faster across the cross-section, which increases flow resistance because of the increasing viscosity due to the temperature decrease [32]. In addition, the solidified surface layers take up more of the flow cross-section than in larger components, making it harder to fill the mold and accurately replicate fine surface details [34]. To address the increase in the viscosity, higher mold temperatures [35] or faster injection speeds [36] are used. However, faster injection speeds, combined with smaller flow cross-sections, require higher pressures during the filling phase [37]. For example, Yokoi et al. [38] observed injection pressures up to 3000 bar at a speed of 1000 mm·s^−1^ over a flow path of 150 mm with a plate thickness of 2 mm. In general, the highest pressure occurs close to the sprue due to the high deformation of the melt by shear and elongation [32]. The pressure decreases throughout the flow path, which creates a pressure gradient [39]. The pressure gradient decreases as the melt flows, resulting in different pressure levels along the flow path [40]. Studies on thin-walled injection-molded parts have shown that uneven pressure distribution during filling leads to noticeable variations in density [41] and thickness [29] along the flow path, regardless of whether dynamic or conventional isothermal mold temperature control is used. The highest density and thickness occur near the gate, where the polymer solidifies under maximum pressure, while at the flow path’s end, the polymer solidifies under minimal pressure influence [28]. Since the viscosity of highly filled material strongly depends on pressure [42], the high injection pressures required for thin-walled molding further increase viscosity, making it even harder to achieve accurate replication and dimensional stability.

In addition to its influence on melt viscosity, hydrostatic pressure also affects the crystallization behavior of semi-crystalline thermoplastics [43]. High-pressure studies on isotactic polypropylene (iPP) have shown that increasing pressure shifts the crystallization temperature to higher values and accelerates the nucleation kinetics, resulting in increased nucleation densities and earlier onset of crystallization compared to ambient pressure conditions [44]. In nanocomposite systems, such as iPP filled with multi-walled carbon nanotubes (MWCNTs), the combined effect of filler-induced nucleation and elevated pressure promotes the formation of the orthorhombic γ-phase instead of the α-phase. Furthermore, the crystallization temperature and nucleation density are further increased, while the spherulite size is reduced compared to the unfilled polymer [45]. Under high-pressure processing conditions, such as injection molding or injection compression molding, solidification may therefore occur earlier not only due to the pressure-induced increase in viscosity but also as a result of pressure-induced crystallization [46]. This premature solidification can lead to an early flow arrest and directly affects the achievable flow path lengths as well as the final morphology of the molded components.

In general, the viscosity can be modeled by a Cross–WLF model, where the Cross model models the shear thinning behavior of the polymer [47] while the influence of the temperature is modeled by the WLF model [48]. The Cross–WLF model incorporates temperature and pressure effects into zero-shear viscosity using WLF shift factors [49]. The Cross model is shown in Equation (1).

(1) $$\eta_{\left(\dot{\gamma }\right)}=\frac{\eta_{0}}{1+{\left(\frac{\eta_{0}}{\tau^{*}}\cdot \dot{\gamma }\right)}^{1-n}}$$

The model contains three parameters where τ* accounts for the critical shear stress, n is the shear thinning coefficient, and *η*_0_ is the value of the viscosity when the shear rate approaches zero. The WLF zero shear viscosity is presented in Equation (2).

(2) $$\eta_{0}(T)=D_{1}exp[\frac{A_{1}(T-D_{2})}{A_{2}+T-D_{2}}]$$

where D_1_, D_2_, A_1_, and A_2_ are coefficients for fitting the temperature dependency. In the specific context of accounting for the pressure effect, the WLF formulation for zero-shear viscosity can be expressed as shown in Equation (3) [50].

(3) $$\eta_{0}(T,P)=D_{1}exp[\frac{A_{1}(T-D_{2}-D_{3}P)}{{\tilde{A}}_{2}+T-D_{2}}]$$

The parameter D_3_ denotes the pressure dependency, while P is the pressure acting on the polymer melt. Modeling the pressure dependency of viscosity requires numerous measurements under pressure, often using advanced and complex equipment [32]. As a result, most material models in simulation software set the pressure coefficient to zero (D_3_ = 0 K∙Pa^−1^) [48], effectively ignoring pressure dependency in these models [51]. When pressure dependency is included, the D_3_ parameter is usually represented as a single constant value. However, this simplification does not capture the actual behavior of thermoplastics, where the D_3_ parameter significantly depends on shear rate [52], temperature [53], and pressure [54], as shown in many studies [50]. Furthermore, most simulation databases do not define a solidification criterion that accounts for the flow stopping due to a pressure-induced exponential increase in viscosity [46]. Instead, flow is usually restricted by a temperature-dependent “no-flow” criterion, corresponding to the crystallization temperature and its pressure-related shift for thermoplastics [55]. This leads to an overestimation of plastic flowability in common simulation tools, even when the D_3_ parameter is applied [50]. In general, the usage of the pressure parameter D_3_ of the seven-parameter Cross–WLF model is recommended by the literature when the following conditions are met [56]:

- The flow path length to wall thickness ratio is bigger than 100;
- The injection pressure exceeds 1.000 bar;
- The wall thickness is lower than 2 mm;
- Using specific materials like PC.

While the influence of the consideration of the pressure dependency with the D_3_ parameter on the flow behavior of unfilled plastic has been studied in the literature [49,50], it has not yet been possible to investigate the influence of the pressure dependence on highly filled plastic compounds in injection molding and injection compression molding. Recent research focuses on modifying the crystal structure of PP and the following optimizing of the mechanical properties of the material [46], while the influence of the pressure-induced crystallization and its effects on the flow behavior of highly filled plastics has not yet been the subject of research. Therefore, the aim of this study is the evaluation of the effect of pressure-dependent viscosity data on the flow behavior of highly filled plastic melts. For this purpose, experiments and simulations were conducted. Based on the material characterization of the compounded material, two Cross–WLF models were fitted. Simulations in Moldex3D were performed both with and without considering the pressure dependency of the material and were validated through experiments. Furthermore, the validated model is used to estimate the maximum achievable flow path length to wall thickness ratios for larger bipolar plates.

## 2. Materials and Methods

### 2.1. Materials

For the experiments, flake-shaped graphite particles (typ: GraphCOND; Georg H. LUH GmbH; Walluf, Germany) with a particle size distribution characterized by a D90 value of 50–70 μm were incorporated into a polypropylene (PP) matrix (type BJ100HP, Borealis AG, Vienna, Austria). The graphite was compounded at a concentration of 80 wt.% using a twin-screw extruder (ZSE HP 27, Leistritz Group, Nuremberg, Germany). Therefore, a dosing speed of 1.0 kg∙h^−1^ for the PP and 4.0 kg∙h^−1^ for the GraphCOND was utilized. The nozzle temperature was set to 270 °C while the screw speed was 150 rpm.

### 2.2. Specimen

**Figure 1:**
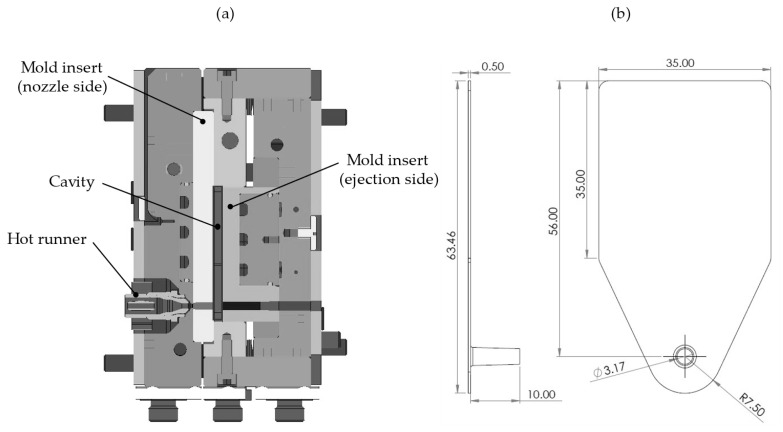
(**a**) Schematic structure of the injection mold; (**b**) plate specimen with the position of the injector point (all dimensions in mm) as previously used by Roth et al. [26].

A simple plate geometry was used to investigate the influence of pressure-dependent viscosity on the results of injection molding and injection compression molding simulations. The injection compression molding process (ICM-DT) and the injection molding process (IM-DT) were performed on an injection molding machine (Arburg Allrounder 370 U, Arburg GmbH + Co. KG, Lossburg, Germany). A schematic overview of the injection mold is provided in Figure 1a, alongside the dimensions of the plate specimens. Figure 1b indicates the positions of the injector point.

The compression stroke was conducted along the machine’s main axis using an embossing frame, which is supported by disc springs on the ejector-side mold plate. This configuration allows for variable adjustment of component thickness between 0.5 mm and 4 mm. Dynamic temperature control was applied within the embossing frame as well as in temperature control cores located near the cavity, both on the nozzle and ejector sides. The system used a dual-circuit pressurized hot- and cold-water system, which included a switchover unit located near the cavity to manage the temperature effectively.

**Figure 2:**
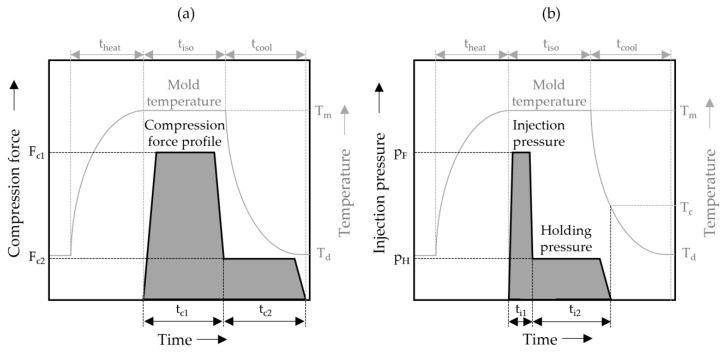
(**a**) Simplified illustration of the schematic multistage ICM-DT process used for the production of the ICM samples. (**b**) Schematic illustration of the used multistage IM-DT process.

The process cycle for the ICM-DT experiments, schematically illustrated in Figure 2a, begins by heating the mold to the target temperature T_m_. Once the desired temperature is reached, the melt is injected into the mold, which is held open by an embossing gap. The material is then shaped into the desired plate geometry using a compression stroke that is both speed- and force-controlled, adjusting to the desired compression forces. Following the molding process, the system enters an isobaric and isothermal holding phase. Finally, the mold is cooled down to the demolding temperature T_d_ while maintaining constant cavity pressure throughout the process. Figure 2b shows the IM-DT process, in which the melt is injected into the cavity at the target temperature T_m_. A holding pressure is then applied at a constant mold temperature, after which the mold is cooled to the demolding temperature T_d_.

In order to investigate the influence of pressure dependence in ICM and IM simulations with dynamic mold control, various mold temperatures were used as the target temperature T_m_, starting with the demolding temperature T_d_ of 80 °C, which corresponds to an isothermal process. The target temperature T_m_ was then increased in steps of 10 °C up to 170 °C, which is the maximum possible temperature with the existing temperature control unit. A constant cycle was selected for these variations, the parameters of which can be seen in Table 1.

While Table 1 outlines the general settings of the dynamic mold temperature control applied in both molding processes, Table 2 summarizes the essential process parameters governing the ICM-DT procedure itself, such as melt temperature, compression stroke, and compression conditions during the various stages of the cycle.

In order to compare the influence of the pressure dependency in the modeling of the viscosity of highly filled compounds, the process sequence was designed as a multistage ICM-DT process. In the first stage, the compression stage, a force of 300 kN is applied in the cavity in order to ensure the flow of the melt. With the pressure relief stage of 50 kN, the flowability of the melt should be restored within the completely filled cavity. The compression speed is limited during both stages to 15 mm·s^−1^ and 5 mm·s^−1^. The parameters for the different stages can be seen in Table 3.

In addition, IM-DT simulations and tests were carried out to compare the influence of the material modeling on the result of the filling processes. The maximum injection pressure of the machine of 2200 bar was selected for filling, while the cavity was filled with a filling time of 1.6 s. After filling, a holding pressure of 1.500 bar was applied for 20 s. Due to the highest possible injection speed selected for the machine, the pressure limit was achieved in every process. This makes it possible to establish comparability between the simulation and the real process. The same parameters as used in the real ICM and IM process were used as settings for the simulation. The hole parameter setting can be seen in Table 4. 

### 2.3. Calculation of the Filling Percentage

To enable a quantitative comparison of the filling degree between the different molded specimens, the degree of filling was calculated based on the measured density of the material and the weight of the individual parts. The weight was determined using a precision balance of type LS 320A SCS (Precisa Gravimetrics AG, Dietikon, Switzerland), while the material density was measured using a gas pycnometer (AccuPyc 1330, Micromeritics GmbH, Unterschleißheim, Germany). Based on three measurements, the material density was determined to be 1.7360 g∙cm^−3^ with a standard deviation of 0.0003 g∙cm^−3^. The theoretical mass of a fully filled specimen was calculated using the part volume obtained from the CAD model in combination with the measured material density.

### 2.4. Thermogravimetric Analysis

The actual filler content of the material was quantified via thermogravimetric analysis (TGA) using a TGA Q5000 instrument (TA Instruments, New Castle, DE, USA) under a nitrogen atmosphere. This approach was selected due to the expected variability in filler concentration arising from potential inaccuracies during the compounding process. The analysis was conducted after the thermal decomposition of the polymer matrix at 500 °C, enabling the quantification of the residual filler. To verify that graphite remained thermally stable at this temperature and that the measurement was not affected by carbonaceous residues originating from the PP matrix, additional control measurements were performed using pure graphite powder and pure PP.

### 2.5. Simulation Software and Settings

For the simulations of the ICM as well as the IM process, Moldex3D 2023 of CoreTech System Co., Ltd. (Zhubei, Taiwan) was used. A five-layer boundary layer mesh (BLM) was applied, with a manually defined boundary layer offset ratio of 3.0 to ensure accurate resolution near the wall regions. The compression zone was discretized using tetrahedral elements, while the runner system was modeled with a structured mesh comprising four inner and five outer layers. A uniform nodal spacing of 0.3 mm was employed throughout the mesh to balance computational efficiency and result accuracy.

### 2.6. Material Model

**Figure 3:**
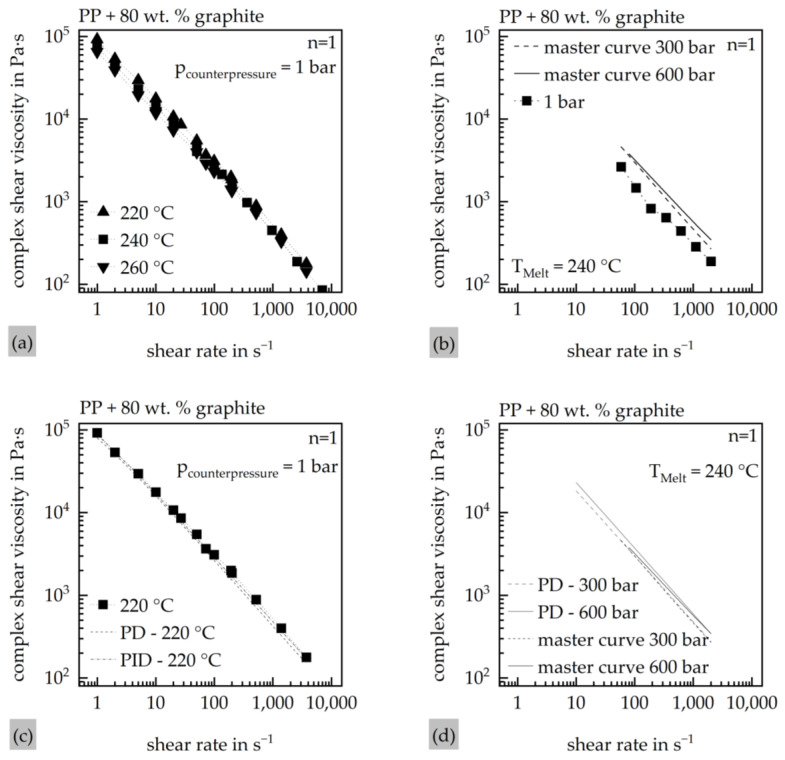
Results of the HCR-CPC measurements of the material. (**a**) Viscosity of the material at certain temperatures with no counterpressure; (**b**) CPC master curve at different counterpressures; (**c**) Cross–WLF fit of both material models at 0 bar and 220 °C melt temperature; (**d**) Cross–WLF fit of both material models at 300 bar and 600 bar at 240 °C melt temperature.

With the results of the material characterization, a material model for the simulations in Moldex3D 2023 was created. The material model was then implemented into the Moldex3D Material wizard as a material card. For the viscosity, two Cross–WLF models were created. For those, the viscosity of the material was measured by a RHEOGRAPH 75 (Göttfert Werkstoff-Prüfmaschinen GmbH, Buchen, Germany), while the fitting of the Cross–WLF models was conducted in the Autodesk Moldflow Data Fitting 2019 Software (Autodesk Inc., San Rafael, CA, USA) using a least-squares optimization. The pressure dependency of the viscosity was tested with the usage of a counterpressure cell (CPC) as well as a connection channel to use the function of the counterpressure viscometer (CPV) of the system. Due to the high viscosity of the material and the associated challenges in its accurate measurement, initial pressure-dependent rheological data were obtained using the CPC. The viscosity model used in the subsequent simulations was based on these measurements. To validate the results obtained from the CPC, complementary measurements were later conducted using the connection channel of the system for the CPV measurements. Since the CPC does not ensure identical shear rates at both the zero-die and the high-pressure die under counterpressure conditions, no correction of the viscosity data was applied in this setup. While the CPV setup enables a Bagley and Weißenberg–Rabinowitsch correction of the pressure-dependent viscosity, such correction was deliberately omitted in this study to maintain comparability between both measurement approaches. The results of the CPC measurements, as well as the fitting of the pressure-independent (PID) and the pressure-dependent (PD) Cross–WLF model on the master curves of the CPC measurements, are shown in Figure 3.

Figure 3a illustrates the material’s shear-dependent viscosity behavior in a double-logarithmic plot, revealing a linear decrease in viscosity with increasing shear rate. Among the influencing factors, shear rate exhibits the most significant impact on viscosity, whereas the influence of temperature appears comparatively minor. In contrast, Figure 3b highlights the pressure dependence of the material: a noticeable increase in viscosity is observed between 0 bar and 300 bar, while just a minor change can be observed between 300 bar and 600 bar. Figure 3c demonstrates that both viscosity models provide a good fit to the experimental data at 0 bar counterpressure and a melt temperature of 220 °C. This fitting quality is further confirmed in Figure 3d, which shows the performance of the pressure-dependent viscosity model (PD) at counterpressures of 300 bar and 600 bar. Both models are shown in Table 5.

**Figure 4:**
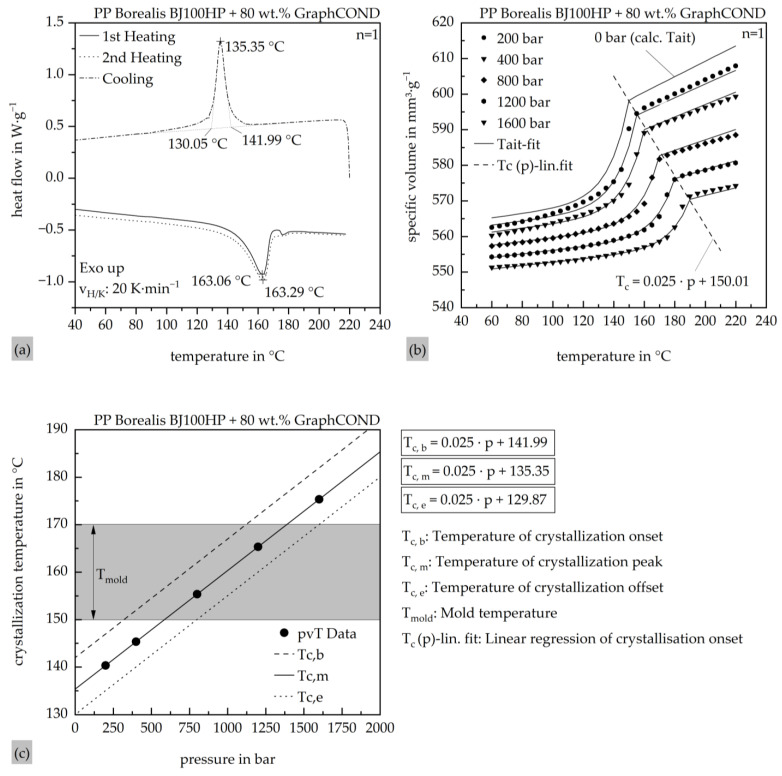
(**a**) Determination of the absolute position of the crystallization range based on DSC measurements; (**b**) determination of the pressure dependence of the crystallization range based on pvT measurements as well as the fit of the calculated pvT modified Tait model; (**c**) pressure- and temperature-dependent process window based on the combined DSC and pvT measurements for the pressure dependence of the crystallization range.

To accurately characterize the crystallization and thermo-rheological behavior of the highly filled polypropylene compound, DSC and pvT measurements were conducted. The DSC analysis was performed using a Discovery DSC2500 (TA Instruments, New Castle, DE, USA), while pvT data were obtained with a RHEOGRAPH 25–50 (Göttfert Werkstoff-Prüfmaschinen GmbH, Buchen, Germany). The corresponding results are shown in Figure 4a,b.

The DSC measurements reveal a crystallization peak at 135.35 °C, with the onset at 141.99 °C and the end at 130.05 °C. Based on this data, the preset Nakamura model in the Moldex3D Material wizard was modified using Netzsch Kinetics Neo software (Netzsch Group, Selb, Germany). The pvT data was used to generate the modified Tait model (2), which was implemented into the Moldex3D material database for accurate description of pressure- and temperature-dependent volume behavior. The calculated Tait model shows good agreement with the measured data across the evaluated pressure and temperature ranges. The exact parameter values used for the Tait model are provided in Table 6.

The thermal properties consisting of the heat capacity and the thermal conductivity of the material were measured in order to complete the material card of the compounded material. The heat capacity was measured with a Discovery DSC2500 (TA-Instruments, New Castle, DE, USA), while the c_p_ baseline adjustment was carried out with a Setaram C80 calorimeter (KEP Technologies, Mougins, France) at a heating rate of 0.1 K∙min^−1^. The thermal conductivity was determined in the melt using a Rheograph 75/25 (Göttfert Werkstoff-Prüfmaschinen GmbH, Buchen, Germany). For the Moldex3D material card, the thermal conductivity and the heat capacity were interpolated using the existing measured values from Table 7.

### 2.7. Setup for Microscopy

To analyze the filler morphology of the molded specimens, selected samples were examined using reflected light microscopy. For this purpose, an Axio Imager.M2 microscope (Carl Zeiss Microscopy Deutschland GmbH, Oberkochen, Germany) was used. Prior to imaging, the samples were embedded in resin and polished to obtain suitable cross-sections.

## 3. Results and Discussion

### 3.1. Filling Content

**Figure 5:**
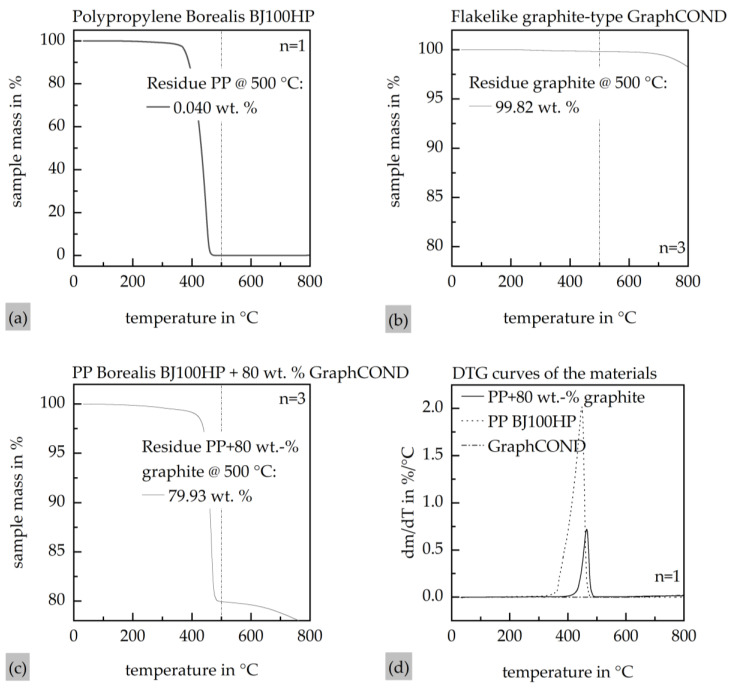
Thermogravimetric analysis with a heat rate of 10 K∙min^−1^ under N_2_ atmosphere. (**a**) Thermogravimetric analysis of the compound; (**b**) thermogravimetric analysis of the pure graphite particles; (**c**) thermogravimetric analysis of the pure PP Borealis BJ100HP; (**d**) DTG curves of the compound as well as the graphite filler and the matrix PP.

Figure 5 presents the results of the thermogravimetric analysis of the compound with a maximum calculated filler content of 80 wt.%. The measurement of the compound exhibits a significant mass loss starting at approximately 400 °C, which is completed at around 500 °C. The average residual mass of the compound determined at 500 °C, *m*c,*res*(500 °C), is 79.93 wt.%, as shown in Figure 5a. To ensure the clarity of the graph, only one measurement curve is shown as an example due to the sufficiently small standard deviation. The measurement results for pure graphite indicate that there is minimal mass loss within the analyzed temperature range. At 500 °C, a residual mass of approximately 99.82 wt.% graphite, *mg*,*res*(500 °C), was determined, as illustrated in Figure 5b. The measurement error due to carbonaceous residues from the degraded PP was quantified by measuring the pure PP matrix material, yielding a residual PP mass content at 500 °C, *mpp*,*res*(500 °C), of approximately 0.040 wt.%, as shown in Figure 5c. Consequently, the filler content in the compound *fc*, used for the ICM-DT experiments, is calculated according to Equation (3) to be 79.71 wt.% and is in good agreement with the set filler content from compounding.

(4) fc=mc,res(500 °C)−mpp,res(500 °C)−(100−mg,res(500 °C))

### 3.2. Filling Behavior of the ICM and IM Process

**Figure 6:**
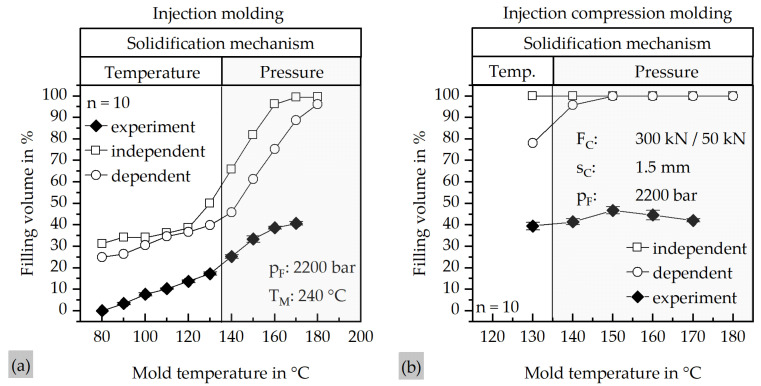
Comparison of the results of the mold filling simulations with the pressure-dependent viscosity model and the pressure-independent viscosity model, as well as the results of the real trials at different mold temperatures. (**a**) Results of the IM process; (**b**) results of the ICM process.

Figure 6 summarizes the results of the simulation and experimental validation for both molding processes. Figure 5a presents the filling behavior obtained from the IM simulations, while Figure 5b displays the corresponding results for the ICM process. In both diagrams, the simulated data is compared with the experimental results from the real molding trials.

Figure 6a illustrates the strong dependence of the filling volume on pressure-dependent viscosity. Without considering the pressure dependency, the simulation model predicts complete filling at approximately 170 °C, whereas with pressure-dependent viscosity, even at 180 °C, complete filling cannot be achieved. The experimental results show a pronounced discrepancy in filling volume compared to the simulations. At mold temperatures below the crystallization temperature of the material, the deviation between simulation and experiment is reduced. This behavior can be explained by the solidification mechanism of the material. At temperatures below 135.35 °C, which represents the crystallization peak, solidification is driven by the mold surface temperature, whereas at temperatures over the crystallization temperature, solidification is governed by pressure.

The similar gradients of all curves below the crystallization temperature indicate that the viscosity of the material is generally underestimated, likely due to particle–particle interactions. Furthermore, the presence of a plateau at higher mold temperatures is evident. Since the mechanism of pressure-dependent solidification is governed by the local pressure in regions with the smallest cross-section, an increase in the global mold temperature has only a marginal effect on the filling volume. This behavior is also observed in the ICM process, where experiments revealed no significant increase in filling volume at higher mold temperatures. Comparable to the IM process, the simulations predict complete filling above a certain temperature, while neglecting the pressure dependency results in simulated filling even at 130 °C. Similarly, the viscosity of the real material is underestimated in the simulations. Interestingly, the experimental filling volume decreases above 150 °C. A possible explanation is the reduced matrix viscosity at elevated temperatures, which may facilitate matrix–filler separation and promote earlier agglomeration of filler particles, thereby hindering further flow.

### 3.3. Increase in Filling Volume with Lower Injection Pressures

**Figure 7:**
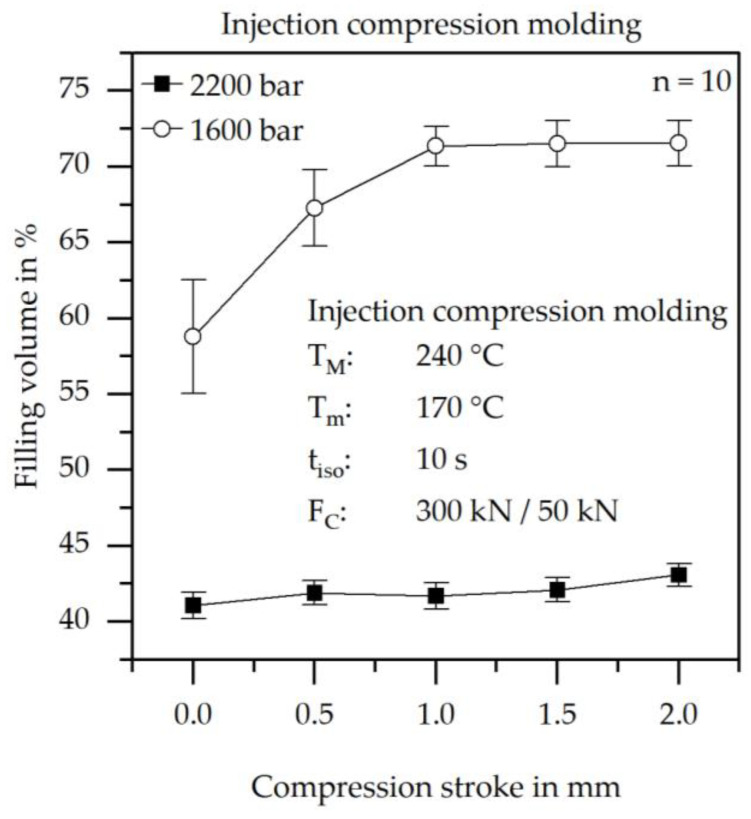
Results of the achieved filling volume at different normal compression strokes and different injection pressures at a constant mold temperature of 170 °C.

To verify the theory of pressure solidification, injection compression molding trials were conducted at different compression strokes with injection pressures of 1600 bar and 2200 bar. The corresponding results are presented in Figure 7.

Figure 7 illustrates the strong dependence of the filling volume on the injection pressure. At an injection pressure of 2200 bar, the maximum filling volume is limited to below 45%, whereas filling volumes exceeding 70% can be achieved at lower injection pressures. An increase in filling volume with increasing compression stroke is also evident. With higher compression strokes, the cavity thickness expands during the filling process. This enlarged cross-section reduces the emerging pressure, thereby improving the achievable flow path length. The nearly constant filling volume over the compression stroke at 2200 bar alongside the steady-state behavior of the 1600 bar curve beyond a compression stroke of 1.0 mm indicates that the limiting factor is found in the cross-section between the sprue and the cavity.

**Figure 8:**
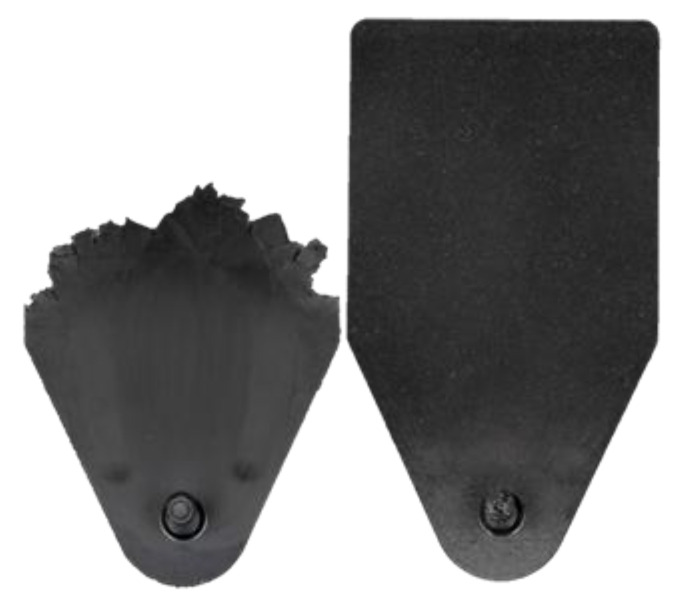
Comparison of a fully filled specimen with a partially filled specimen with 1600 bar injection pressure and 0.0 mm compression stroke from Figure 7.

Figure 8 shows the difference between a partially filled specimen with 1600 bar injection pressure and a 0.0 mm compression stroke from Figure 7 and a fully filled specimen. The fully filled specimen was injection compression molded in advance with a material with a lower filling degree.

**Figure 9:**
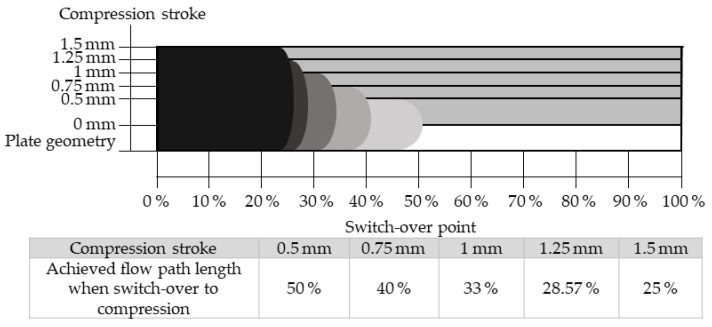
Schematic representation of the melt and its flow front at different compression strokes after the injection process. The switch-over point indicates the flow length reached after injection as a percentage of the total flow path of the part. Due to volume constancy, which is independent of the compression stroke, the switch-over point decreases with increasing compression stroke.

Figure 9 depicts the cavity cross-section at different compression strokes along with the corresponding melt front positions at the compression-stroke-dependent switchover points.

With increasing compression stroke, the melt front reaches shorter flow path percentages at the switchover point. Owing to the extended geometry, the pressure in both the cavity and the transition area from the sprue to the cavity decreases, as these regions are enlarged by the compression stroke. As a secondary effect, the melt remains more compact at higher compression strokes, resulting in a lower surface-to-volume ratio. Consequently, the melt cools less rapidly to the mold surface temperature, retains a higher temperature, and therefore exhibits a lower viscosity.

### 3.4. Morphology Formation

**Figure 10:**
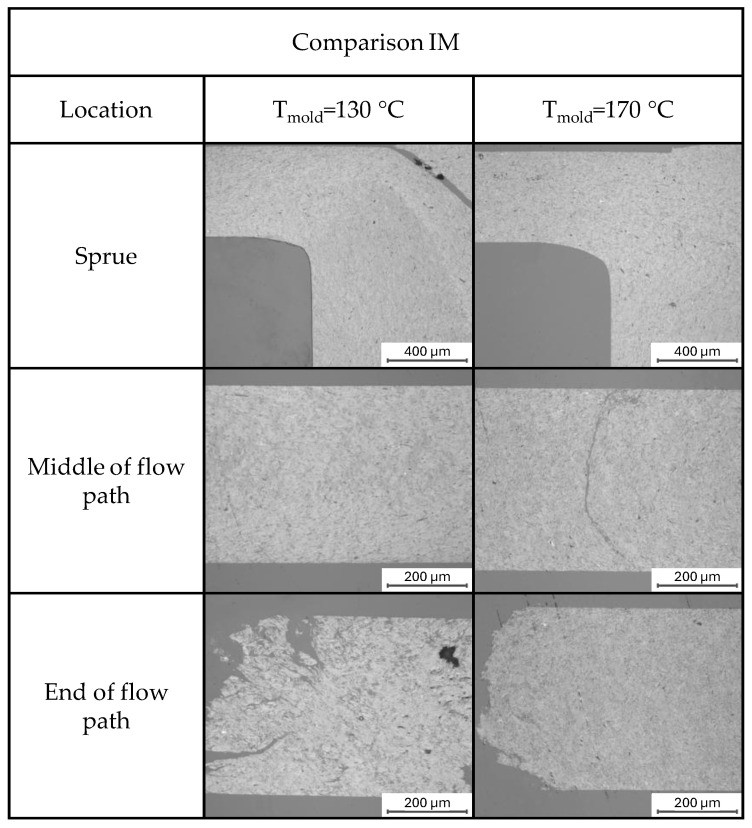
Reflected light microscopy images of polished cross-sections from injection-molded specimens, comparing two mold temperature settings: 130 °C (left column) and 170 °C (right column). Images were taken at three locations along the flow path: near the sprue, in the middle of the flow path, and at the end of the flow path. The micrographs illustrate the influence of mold temperature on the internal morphology and structure development during molding.

Microscopically observed morphologies obtained by reflected light microscopy are shown in Figure 10. The sprue images exhibit locally varying morphological structures that depend on the applied mold temperature. Considering a mold temperature of 130 °C, solidification occurs immediately upon contact with the mold surface. The intrinsically high thermal conductivity of the filled material accelerates the locally occurring heat transfer, and the solidified layer constricts the cross-section available to the subsequent melt. As a result, the attainable filling time is limited by gate freezing. Once the gate has solidified, no additional material enters the cavity and the fillers compact, evident as darker grey regions in the material. Contrasting reduced mold temperatures, an elevated mold temperature of 170 °C contributes to the formation of an increased morphological homogeneity. With the mold held above the crystallization temperature, the material does not solidify at the wall, permitting flow across the full cross-section of the cavity. The particles remain evenly distributed in the matrix, with no evidence of agglomeration or particle damage. In the mid-flow region at 130 °C, the reduced cross-section yields a more compacted particle arrangement. In contrast, a mold temperature of 170 °C promotes a predominantly homogeneous distribution. At the end of the flow path, the flow front appears rough, particularly at reduced mold temperatures of 130 °C. The porous zone near the end of the flow path measures roughly 100 μm at 170 °C, but exceeds 600 μm at 130 °C, associated with the formation of several matrix-rich areas. This pattern indicates a significant influence of particle–matrix separation at the end of the flow path due to the low-pressure stage, likely associated with the influence of an increased matrix viscosity under accelerated cooling. At 130 °C, the flow front arrests when the gate solidifies, whereas at 170 °C, filling ceases when the pressure surpasses the threshold for pressure-dependent crystallization.

In general, it can be observed that the higher mold temperature enabled a better flow of the compound since mold temperatures over the crystallization temperature do not create a solidified material layer, which decreases the cross-section of the cavity.

**Figure 11:**
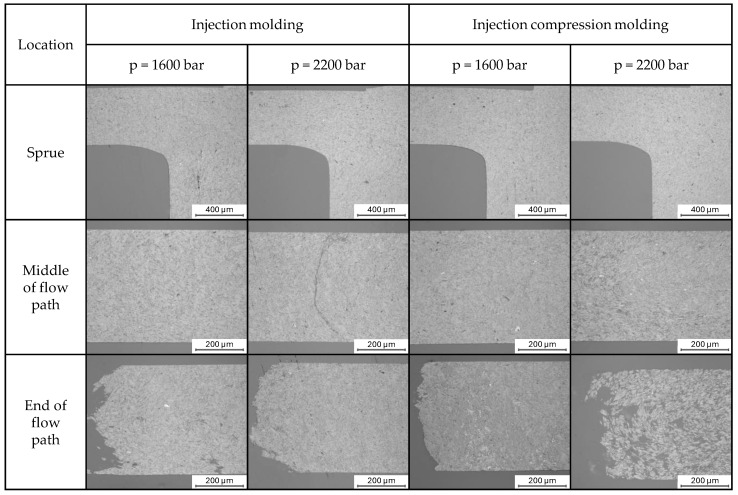
Reflected light microscopy images of polished cross-sections from injection-molded (**left**) and injection compression-molded (**right**) specimens at two different packing pressures (1600 bar and 2200 bar). Images were taken at three positions along the flow path: near the sprue, in the middle of the flow path, and at the end of the flow path. The direction of the flow path is from the right to the left. The micrographs illustrate the influence of the molding process and the packing pressure on the internal filler morphology.

Macrographs of formed melt front topologies dependent on applied injection pressures are shown in Figure 11.

For the sprue area, a compaction of the fillers can be observed with higher injection pressures as well as the addition of a compression force in the ICM process. In the middle of the flow path, no orientation of the fillers can be detected. The apparent crack at the IM sample with 2200 bar is attributable to the manual preparation process involving grinding. The samples do not show a closed flow front, with individual cracks running perpendicular to the flow direction in some areas. During the grinding process, the surface was accidentally polished in such an area. For the specimen produced by the ICM process at an injection pressure of 2200 bar, a distinct transition zone can be identified. This zone is characterized by a matrix-rich filler distribution in regions remote from the gate, followed by a sharp transition into an area with highly compacted fillers. The underlying mechanism can be attributed to the continuously decreasing flow-front velocity along the constant cavity cross-section, which prevents the establishment of a steady-state flow. As a result, the advancing melt front experiences increasing compression by subsequently injected, highly viscous material. This progressive compression raises the pressure in the sprue and gate over the course of injection, which in turn recursively alters the melt viscosity and the particle–particle interactions. Based on previously discussed experimental and numerical findings, this feedback loop produces a self-intensifying effect that ultimately triggers the pressure-dependent solidification of the melt. The matrix-rich region on the left side of the specimen can be explained by a melt breakthrough occurring during filling. In this region, the melt shows no contact with the cavity wall, as evidenced by the absence of a characteristic flat wall-imprinted surface structure at the flow edges. This melt breakthrough was not observed under other processing conditions, indicating that it results from the combined effect of the increased effective wall thickness inherent to the ICM process and the elevated injection pressure of 2200 bar. Once the runaway effect initiates, filler agglomeration begins at the position of the former melt front, which maintained continuous wall contact due to the flat surface of the specimen. This observation is consistent with the pressure-driven solidification behavior described above.

### 3.5. Comparison of the Measured Viscosity with a CPV and a CPC at Different Pressure Stages

**Figure 12:**
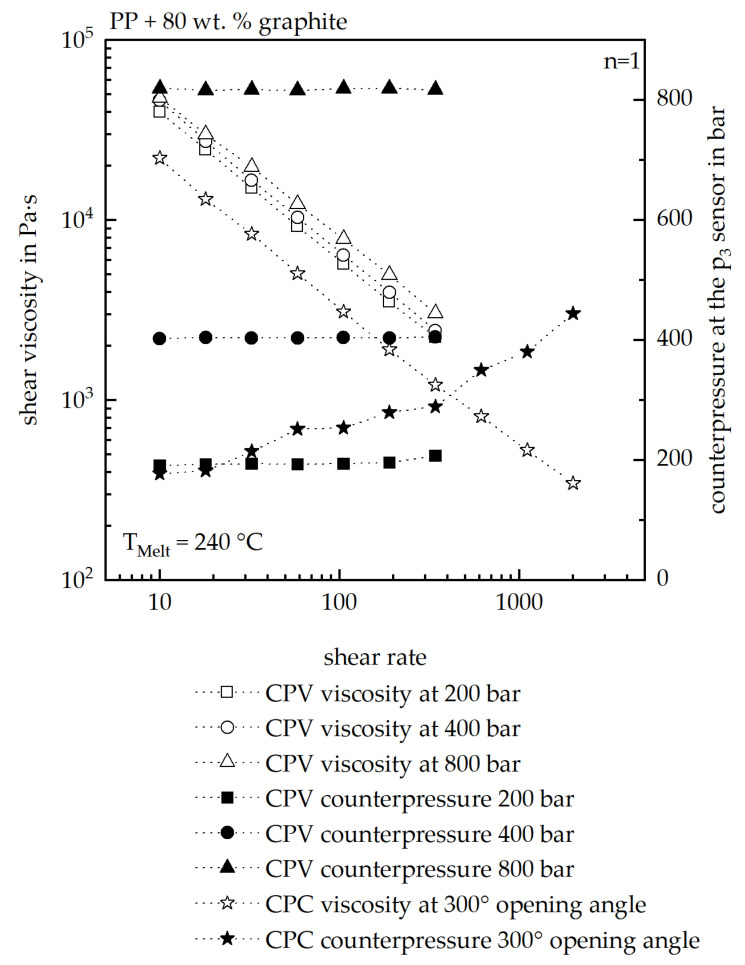
Comparison of the measured viscosities as well as the measured counterpressures at the p_3_ sensor with the usage of the CPC as well as the CPV. For the CPC, a master curve was then fitted out of the measured curves. A melt temperature of 240 °C was used for the comparison.

Figure 12 presents a comparison of the measured viscosity curves as well as the measured counterpressure value at the p_3_ sensor of the HCR of the investigated material.

Overall, the CPV measurements show a clear dependency of the viscosity on the counterpressure at the p3 sensor. The counterpressure can be controlled with high precision in this setup. In contrast, during the CPC measurements, the counterpressure varies continuously, requiring the construction of a master curve from the acquired dataset. For this purpose, the viscosity values are first grouped according to their corresponding pressure levels and subsequently shifted to a selected reference curve. This procedure enables the generation of a master curve; however, it inherently introduces deviations both in the resulting master curve and in the fitted rheological model.

As all viscosity models were derived from CPC measurements, the simulation results underestimate the pressure-dependent viscosity, as already demonstrated in Figure 6. This highlights the necessity of using CPV measurements to reliably determine the pressure-dependent viscosity of highly filled polymers, thereby avoiding underestimation in simulations. The accuracy of simulation models based on CPV data hence needs to be evaluated in future studies.

## 4. Conclusions

Based on combined numerical and experimental investigations on both the compression injection molding and the injection molding of graphite-filled polypropylene, the present study provides an overview of pressure-induced effects with a particular focus on the self-intensifying pressure-induced solidification of highly filled thermoplastics under elevated pressures. During injection molding, experimental observations indicate a considerable overestimation of the obtained degree of filling during pressure-dependent numerical simulations. Despite the consideration of pressure-dependent effects in state-of-the-art WLF modeling, the processing of exemplary graphite-filled systems showed a runaway solidification, which we hypothesize to lead to local pressure excursions upon the initial, merely localized solidification of the melt. Similarly, the transfer to injection compression molding displays a considerable divergence between WLF-based numerical modeling and experimental observations. The effect of runaway pressure excursions can be observed in a significant negative correlation of injection pressure during injection compression molding and the corresponding degree of filling. While experimental observations under reduced pressures show filling degrees similar to numerical observations, elevated injection pressures fail to obtain similar filling degrees, showing a significant reduction. These observations imply a considerable influence of the injection step on the intermediary filling of the mold prior to the compression step. These interpretations are supported by microscopic investigations that show morphological variations during injection compression under high injection pressures, indicating the influence of both an intermediary solidification as well as a reduced extent of stress relaxation under increased injection pressures. Thus, the observations imply the requirement for the consideration of the pvT-derived solidification line in future numerical simulations, allowing for implicitly considering the effect of solidification-induced pressure excursions on the flow regime of both unfilled and filled thermoplastics. In general, despite the identified solidification-related limitations, the use of a pressure-dependent Cross–WLF model based on viscosity data obtained through CPV can be recommended for simulation of highly filled plastics.

## Figures and Tables

**Table 1:** Parameters of the dynamic mold control process for the ICM as well as the IM process.

Parameter (Unit)	Value
Mold temperature T_m_ (°C)	80–170
Demolding temperature T_d_ (°C)	80
Heating time t_heat_ (s)	125
Isothermal time t_iso_ (s)	12
Cooling time t_cool_ (s)	110
Plate thickness d	0.5

**Table 2:** Main process parameters of the production of the ICM-DT process.

Parameter (Unit)	Value
Melt temperature T_M_ (°C)	240
Compression stroke s_c_ (mm)	1.5
Filling time (s)	1.6
Compression speed v_c_ (mm·s^−1^)	15/5
Compression force F_c_ (kN)	300/50
Injection speed (ccm·s^−1^)	540
Screw diameter (mm)	18

**Table 3:** Parameter settings for both stages of the compression process of the ICM process. The existing cavity pressure shows the resulting pressure with the applied compression force.

Parameter (Unit)	Value—Stage c1	Value—Stage c2
Duration of the phase t_c_ (s)	5	5
Compression force Fc (kN)	300	50
Compression speed v_c_ (mm·s^−1^)	15	5
Existing cavity pressure (bar)	1606	267

**Table 4:** Parameter settings of the injection molding process with isothermal (iso) and dynamic mold temperature control (DT).

Parameter (Unit)	Value
Melt temperature T_M_ (°C)	240
Filling time (s)	1.6
Holding pressure time (s)	20
Injection pressure p_F_ (bar)	2200
Holding pressure p_H_ (bar)	1500
Injection speed (ccm·s^−1^)	540
Screw diameter (mm)	18

**Table 5:** Values of the models used for the viscosity. Model PID neglects the effect of the pressure-dependent viscosity, while Model PD accounts for the effect.

Parameter	Model PID—Independent	Model PD—Dependent	Unit
n	0.22	0.2029	-
τ*	65,948.9	77,621.6	Pa
D_1_	1.07225 × 10^24^	2.0408 × 10^20^	Pa∙s
D_2_	263.15	263.15	K
D_3_	0	2.628 × 10^−7^	K∙Pa^−1^
A_1_	51.58	41.666	-
Ã_2_	51.6	51.6	K

**Table 6:** Modified Tait model (2) of the compounded material.

Parameter	Value	Unit
b_1L_	5.984 × 10^−4^	m^3^∙(kg)^−1^
b_2L_	2.166 × 10^−7^	m^3^∙(kg∙K)^−1^
b_3L_	1.90609 × 10^8^	Pa
b_4L_	3.461 × 10^−3^	K^−1^
b_1S_	5.704 × 10^−4^	m^3^∙(kg)^−1^
b_2S_	5.769 × 10^−8^	m^3^∙(kg∙K)^−1^
b_3S_	4.5811 × 10^8^	Pa
b_4S_	7.124 × 10^−4^	K^−1^
b_5_	423.14	K
b_6_	2.48 × 10^−7^	K^−1^
b_7_	2.798 × 10^−5^	K
b_8_	0.07878	K∙(Pa)^−1^
b_9_	2.423 × 10^−8^	m^3^∙(kg)^−1^

**Table 7:** Thermal properties of the material as tabulated data for the material model.

Temperature (°C)	Heat Capacity c_p_ (J∙(kg∙K)^−1^)	Thermal Conductivity λ (W∙(m∙K))^−1^
100	1339.2	/
110	1414.8	/
120	1512	/
130	1711.2	/
140	3940.8	/
150	1731.6	/
160	1560	6.396
170	1566	/
180	1592.4	6.498
190	1627.2	/
200	1665.6	6.718
210	1711.2	/
220	/	6.595

## Data Availability

The data presented in this study are available upon request from the corresponding author. The data are not publicly available due to confidentiality reasons.

## Abbreviations

The following abbreviations are used in this manuscript:

ICM	Injection compression molding
CPC-HCR	Coupled counterpressurized high-pressure capillary rheometry
WLF model	Williams–Landel–Ferry model
IM	Injection molding
PEMFC	Polymer electrolyte membrane fuel cell
TMCs	Transition metal carbides
CNTs	Carbon nanotubes
PP	Polypropylene
PPS	Polyphenylene sulfide
DT	Dynamic mold control
ICM-DT	Injection compression molding with dynamic mold control
S/V	Surface-to-volume ratio
PC	Polycarbonate
IC-DT	Injection molding with dynamic mold control
MWCNTs	Multi-walled carbon nanotubes
T_m_	Mold temperature
T_d_	Demolding temperature
t_heat_	Heating time
t_iso_	Isothermal time
t_cool_	Cooling time
d	Plate thickness
T_M_	Melt temperature
s_c_	Compression gap
v_c_	Compression speed
F_c_	Compression force
t_c_	Phase duration
p_f_	Injection pressure
p_h_	Holding pressure
TGA	Thermogravimetric analysis
BLM	Boundary layer mesh
CPC	Counterpressure cell
CPV	Counterpressure viscosimeter
PID	Pressure-independent viscosity model
PD	Pressure-dependent viscosity model
DSC	Dynamic differential calorimetry
PVT	Pressure–volume–temperature measurement
T_c,b_	Temperature of crystallization onset
T_c,m_	Temperature of crystallization peak
T_c,e_	Temperature of crystallization offset
c_p_	Heat capacity
λ	Thermal conductivity
m_c/pp/g,res_	Residual mass of the compound/polypropylene/graphite
f_c_	Filling content
